# Analysis of clinical uncertainties by health professionals and patients: an example from mental health

**DOI:** 10.1186/1472-6947-9-34

**Published:** 2009-07-10

**Authors:** Keith Lloyd, Matteo Cella, Michael Tanenblatt, Anni Coden

**Affiliations:** 1 Institute of Life Science, School of Medicine, Swansea University, Swansea, SA2 8PP, UK; 2 IBM T.J. Watson Research Center 19 Skyline Drive, P.O. Box 704 Hawthorne, NY 10532, USA

## Abstract

**Background:**

The first step in practising Evidence Based Medicine (EBM) has been described as translating clinical uncertainty into a structured and focused clinical question that can be used to search the literature to ascertain or refute that uncertainty. In this study we focus on questions about treatments for schizophrenia posed by mental health professionals and patients to gain a deeper understanding about types of questions asked naturally, and whether they can be reformulated into structured and focused clinical questions.

**Methods:**

From a survey of uncertainties about the treatment of schizophrenia we describe, categorise and analyse the type of questions asked by mental health professionals and patients about treatment uncertainties for schizophrenia. We explore the value of mapping from an unstructured to a structured framework, test inter-rater reliability for this task, develop a linguistic taxonomy, and cross tabulate that taxonomy with elements of a well structured clinical question.

**Results:**

Few of the 78 Patients and 161 clinicians spontaneously asked well structured queries about treatment uncertainties for schizophrenia. Uncertainties were most commonly about drug treatments (45.3% of clinicians and 41% of patients), psychological therapies (19.9% of clinicians and 9% of patients) or were unclassifiable.(11.8% of clinicians and 16.7% of patients). Few naturally asked questions could be classified using the well structured and focused clinical question format (i.e. PICO format). A simple linguistic taxonomy better described the types of questions people naturally ask.

**Conclusion:**

People do not spontaneously ask well structured clinical questions. Other taxonomies may better capture the nature of questions. However, access to EBM resources is greatly facilitated by framing enquiries in the language of EBM, such as posing queries in PICO format. People do not naturally do this. It may be preferable to identify a way of searching the literature that more closely matches the way people naturally ask questions if access to information about treatments are to be made more broadly available.

## Background

When practising evidence-based medicine (EBM), the ability to translate clinical queries into structured and focused clinical questions has been described as the first step of evidence based practice[1,2]. In this way, it is argued, that answers can be found where evidence exists from systematic reviews and clinical trials. If the clinical question cannot be answered by referring to the evidence base then an uncertainty has been identified. The well built clinical question is seen as the key to finding the research evidence and making evidence based decisions. Teachers of EBM contend that the well structured clinical question contains four elements within it. These are the Patient, the Intervention, the Comparison and the Outcome [3]. Together these elements form the acronym PICO. It is argued that the PICO format makes it relatively straightforward to elicit and combine the appropriate terms needed to find the evidence when searching databases such as PubMed [4]. The PICO framework has been adopted by the U.S. National Library of Medicine, which with its over 16 million citations forming MEDLINE, is the largest and most used web source of abstracts. However, the ability to ask PICO style questions, search for the literature effectively and interpret the search results is not intuitive and requires training [5]. Previous studies have looked at how effectively physicians ask clinical questions [6]. This issue is becoming applicable to the general public, which is increasingly turning to internet resources to address uncertainties they might have about treatments.

In this study we focus on questions about treatments for schizophrenia posed by mental health professionals and patients. The objective was to gain a deeper understanding about the types of the questions which are asked naturally and whether they could be reformulated in a structured PICO framework. Furthermore, we defined a question-taxonomy and evaluated whether certain categories within a taxonomy lend themselves better for formulating well defined (and unambiguous) queries. The ultimate goal is to then devise a system within which to channel a user to pose good queries.

## Methods

A convenience sample of patients and clinicians recruited from a web-survey, a conference and members of a mental health charity were asked to write down in their own words an uncertainty or question they had about the effect of treatment for schizophrenia. Both the form and the web-survey gave a brief explanation of uncertainty and examples of types of treatment uncertainties No ethical issues were identified in this non experimental, observational study conducted on anonymous responses to a survey. In order to evaluate the presence and the accuracy of the PICO elements present in peoples' responses, two of us (AC and MT) independently rated the questions for the presences of PICO elements. Results were then compared, discrepancies identified and discussed to agree on definitions. We then assessed inter-rater agreement. Every question was analysed and searched for PICO elements (Patients, Intervention, Comparison and Outcome) and once an element was found, appropriate text corresponding to the element was highlighted. We then examined the questions for evidence of a linguistic taxonomy. Questions were clustered according to an emergent taxonomy and associations between these categories and PICO elements were explored. Definitions and examples of the linguistic taxonomy are given in the results section.

## Results

### Respondents and their questions

489 questions were collected for the Database of Uncertainties about the Effect of Treatment (DUET) for mental health from various health professionals and patients [7]. For the purpose of this exercise a subset of 239 questions on schizophrenia was selected, 161 from clinicians and 78 from patients. All 239 questions about schizophrenia were grouped according to the topic of enquiry. Twelve topics were identified. There was a good deal of similarity between the topics selected by clinicians and patients. Both groups were most likely to ask questions about drug therapies, followed by psychological therapies, followed by questions or statements that were not classifiable (Table 1).

**Table 1:** Frequency of topics raised by clinicians and patients. Percentage in brackets refers to the within group frequency.

Topic	Clinician n (%)	Patient n (%)
Drug	73 (45.3%)	32 (41%)

Psychological therapies	32 (19.9%)	7 (9%)

Unclassified	19 (11.8%)	13 (16.7%)

Diagnosis	8 (5%)	7 (9%)

Service delivery	8 (5%)	5 (6.4%)

Device	7 (4.3%)	3 (3.8%)

Social care	4 (2.5%)	4 (5.1%)

Complementary therapy	3 (1.9%)	3 (3.8%)

Education & training	2 (1.2%)	3 (3.8%)

Exercise	2 (1.2%)	0 (0%)

Environment	1 (0.6%)	1 (1.3%)

Stigma	1 (0.6%)	0 (0%)

Research	1 (0.6%)	0 (0%)

Total	161 (100%)	78 (100%)

### PICO elements in patient and clinician uncertainties

The most common PICO element identifiable in the questions asked by both patients and clinicians was an "Intervention" followed by an "Outcome" followed by a "Patient". The PICO element least likely to be present was a "Comparator" (Table 2).

**Table 2:** PICO elements identified as present in the uncertainties asked by patients and clinicians about schizophrenia.

			Pico	Elements	
		
		Patient	Intervention	Comparison	Outcome
Rater 1 (AC)	Clinician n (%)	60 (37.3%)	107 (66.5%)	18 (11.2%)	19 (11.8%)
	
	Patient n (%)	18 (23.1%)	40 (51.3%)	3 (3.8%)	3 (3.8%)

Rater 2 (MT)	Clinician n (%)	64 (39.8%)	106 (65.8%)	19 (11.8%)	89 (55.3%)
	
	Patient n (%)	31 (39.7%)	46 (59%)	6 (7.7%)	41 (52.6%)

Kappa (k) for Inter-Rater Reliability	Clinician k (p)	0.79 (p < 0.05)	0.63 (p < 0.05)	0.54 (p < 0.05)	0.06 (p > 0.05)
	
	Patient k (p)	0.39 (p < 0.05)	0.64 (p < 0.05)	0.18 (p < 0.05)	0.09 (p < 0.05)

Inter-rater agreement on the PICO elements contained in clinicians' questions and separately for patient questions as calculated by Cohen's Kappa coefficient [8] are also shown in table 2. Kappa coefficients show moderate agreement between the raters for clinician and patient questions.

### Identifying a linguistic taxonomy

To identify the structure and content of the questions asked by respondents' we carried out a syntactic analysis to derive a taxonomy of question types. This exercise was conducted for the clinician questions. Patient generated questions were omitted for two reasons. Firstly, there was low inter-rater agreement for identifying PICO elements in patient generated questions. Secondly, because there were smaller number for patients the assumptions underlying the chi square test were not valid. Linguistic analysis of elements naturally occurring within clinicians' questions about treatment uncertainties for schizophrenia were classified into the following categories: Comparison queries, How queries, ill-defined queries, what queries, why queries, when queries and statements. These categories are summarised in Table 3 together with examples from each category.

**Table 3:** Linguistic analysis of elements naturally occurring within clinicians, and patients, questions about treatment uncertainties for schizophrenia

Response Class	Definition	Example
Comparison query	The desired answer is to state the similarities and differences between two or more concepts or events.	"Old versus. atypical antipsychotic"

How query	The desired answer explains a mode of action, mechanism or process.	"How to access the best counselling support"

What query	The desired answer is a particular concept or event.	What is the difference between depression and happiness?

When query	The desired answer is a time point.	"When do they use E.C.T. treatment?"

Why Query	The desired answer explains the cause of a concept or event.	"Why is E.C.T. banned in several countries?"

Statement	A query is categorized as a statement, if it is an opinion.	"Too much medication is not regularly reviewed"

Ill defined query	Not categorized in any of the previous response classes.	"Does ECT work?"

### Comparing Syntactic classes with well structured clinical questions in PICO format

Table 4, shows associations between PICO elements and the linguistic categories we had generated. Naturally occurring questions that involved "what" type queries were the ones most likely to contain identifiable PICO elements (x^2 ^= 33.31 p < 0.0001). Strong associations were observed for I (x^2 ^= 27.52, p < 0.0001) and O (x^2 ^= 23.97 p = 0.001). The statement category of query was the least likely to contain PICO elements.

**Table 4:** Relationship between PICO elements and the linguistic categories contained in clinicians' questions (n = 161) about treatment uncertainties for schizophrenia:

	Number and Percentage (%) of questions in which PICO element is present			
**Response Class**	**Patient**	**Intervention**	**Comparison**	**Outcome**

Comparison query	12 (17.9%)	19 (17.6%)	7 (35%)	16 (15.8%)

How query	14 (20.9%)	16 (14.8%)	0 (0%)	15 (14.9%)

What query	24 (35.8%)	33 (30.65)	3 (15%)	34 (33.7%)

When query	5 (7.5%)	7 (6.5%)	2 (10%)	5 (5%)

Why Query	3 (4.5%)	7 (6.5%)	2 (10%)	7 (6.9%)

Statement	5 (7.5%)	10 (9.3%)	4 (20%)	13 (12.9%)

Ill-defined query	4 (6%)	16 (14.8%)	2 (10%)	11 (10.9%)

Total	67 (41.6%)	108 (67.1%)	20 (12.4%)	101 (62.7%)

## Discussion

We asked mental health patients and clinicians to identify an uncertainty about the treatment of schizophrenia. Clinicians and patients asked similar types of questions. Few naturally asked structured and focused clinical questions in the PICO format. However, the team was often able to identify PICO elements present in patient and clinicians questions. In many questions those PICO elements were not present making questions hard to classify using the structured clinical question format from evidence based medicine. Other taxonomies may better capture the nature of questions people naturally ask, for example a linguistic taxonomy of the type described here. Access to EBM resources is greatly facilitated by framing enquiries in the language of EBM [9,10]. People do not naturally do this. It may be preferable to identify a way of searching the literature that more closely matches the way people naturally ask questions if access to information about treatments are to be made more broadly available [11].

The main limitation of our study is that this is a secondary analysis of data obtained using a convenience sample from one disease area. Our findings may or may not generalise to other patient and clinician groups. However, if anything, we would expect those persons who took the time to reply to our survey to be among the more interested ones in evidence based practice than those who did not. Respondents were deliberately not asked to structure their responses using the PICO format so as to minimise response bias.

## Conclusion

It is basic principle of EBM that translating clinical uncertainty into structured and focused clinical questions is a prelude to searching the literature. Much time and effort is spent teaching clinicians to ask questions in this PICO format. Our results support the view that people do not naturally ask questions in a well structured format. Broadening access to EBM resources will either require training everyone, both patients and clinicians, in EBM or providing them with ready digested answers to common uncertainties and queries or ideally by identifying a method of searching the literature which does not require the searcher to be trained in EBM [12,13]. Such an approach would greatly broaden access to knowledge and information about health care. It remains to be seen whether PICO elements result in better outcomes compared to other question formulations. Natural language queries or specific typologies of question might be more accurate in retrieving relevant.

## Competing interests

The authors declare that they have no competing interests.

## Authors' contributions

KL conceived the study, and participated in its design and coordination. KL and MC carried out the original data collection. All authors conceived and carried out the data analysis and interpretation. All authors read and approved the final manuscript.

## Pre-publication history

The pre-publication history for this paper can be accessed here:

http://www.biomedcentral.com/1472-6947/9/34/prepub
